# Dataset about the adoption of winter cover crops at the municipality level for mainland France

**DOI:** 10.1016/j.dib.2022.108544

**Published:** 2022-08-17

**Authors:** Benjamin Nowak, Audrey Michaud, Gaëlle Marliac

**Affiliations:** a Université Clermont Auvergne, AgroParisTech, INRAE, VetAgro Sup, UMR Territoires, 63370, Lempdes, France; b Université Clermont Auvergne, INRAE, VetAgro Sup, UMR Herbivores, 63122, Saint-Genès-Champanelle, France; c Université Clermont Auvergne, INRAE, UMR GDEC, 63000, Clermont-Ferrand, France

**Keywords:** Cover crops, Nutrient leaching, Agriculture, Carbon sequestration, Soil erosion, Farming

## Abstract

Winter soil cover by vegetation is associated with multiple benefits, such as increasing soil carbon storage and reducing erosion and nutrient leaching. This dataset provides an estimate of winter soil cover before spring-sown crops at municipality level for mainland France for two years (2018 and 2019). These estimates were obtained through the monitoring of all plots with spring-sown crops, declared within the context of the European Common Agricultural Policy. Detection of plots with winter soil cover was achieved through the analysis of Normalized Difference Vegetation Index (NDVI) time series, computed from Sentinel-2 multispectral images.

For this dataset, it was considered that soil cover had to exceed 50% for a plot to be considered as covered by vegetation. Based on the literature, this corresponds to a threshold NDVI value between 0.45 and 0.59. To allow for sensitivity and uncertainty analyses for future studies that may be conducted using these data, three estimates of winter soil cover are given: minimum (based on the cultivated area exceeding the upper NDVI threshold of 0.59), maximum (considering the lower NDVI threshold of 0.45) and best estimate (mean NDVI threshold of 0.52). This dataset may be useful primarily to researchers working on biogeochemical cycle modeling or to government agencies, as several public policies (such as the Nitrates Directive) aim at developing winter cover crops.

**Specifications Table**

**Table:** 

Subject	Agronomy and Crop Science
Specific subject area	Evaluation of the adoption of winter cover crops before spring-sown for mainland France
Type of data	Table (csv format)
How the data were acquired	Large scale analysis of Normalized Difference Vegetation Index (NDVI) computed from Sentinel-2 multispectral images with the Google Earth Engine platform
Data format	Analyzed
Description of data collection	Selection of all plots with spring crops for 2018 and 2019 with the French Registre Parcellaire Graphique. Computation of Normalized Difference Vegetation Index (NDVI) time series for each plot. Detection of plots with winter soil cover using thresholds defined in the literature.
Data source location	Country scale: mainland France Raw data sources used: - French Registre Parcellaire Graphique to defined plot boundaries (available here: https://www.data.gouv.fr/fr/datasets/registre-parcellaire-graphique-rpg-contours-des-parcelles-et-ilots-culturaux-et-leur-groupe-de-cultures-majoritaire/) - Sentinel-2 multispectral images to compute Normalized Difference Vegetation Index (NDVI) time series for each plot. (available here: https://scihub.copernicus.eu/dhus/#/home)
Data accessibility	Repository name: WinterSoilCover (https://github.com/BjnNowak/WinterSoilCover) Data identification number (DOI): 10.5281/zenodo.6822857 Direct URL to data: https://raw.githubusercontent.com/BjnNowak/WinterSoilCover/main/dataset/dataset_winter_soil_cover.csv Direct URL to code: https://github.com/BjnNowak/WinterSoilCover/blob/main/code/gee_code_example Instructions for accessing these data: None (open access related research article: B. Nowak, G. Marliac, A. Michaud, Estimation of winter soil cover by vegetation before spring-sown crops for mainland France using multispectral satellite imagery, Environ. Res. Lett. 16:064,024 (2021). https://doi.org/10.1088/1748-9326/ac007c)
Related research article	B. Nowak, G. Marliac, A. Michaud, Estimation of winter soil cover by vegetation before spring-sown crops for mainland France using multispectral satellite imagery, Environ. Res. Lett. 16:064,024 (2021). https://doi.org/10.1088/1748-9326/ac007c

**Value of the Data**

• Winter soil cover by vegetation is associated with multiple benefits, such as increasing soil carbon storage and reducing erosion and nutrient leaching.
• This dataset may be useful primarily to researchers working on biogeochemical cycle modeling or to government agencies.
• Estimating the adoption of winter cover crops can help assess the additional soil carbon storage provided by these crops.
• As the winter period is a critical period for leaching, the estimation of winter soil cover is essential for models aiming to evaluate the transfer of mineral elements to waterways.
• As several public policies (such as the Nitrates Directive) aim at developing winter cover crops, this dataset will allow to evaluate the efficiency of these policies.

## 1 Data Description

Dataset For each municipality and for each spring crop of mainland France, this dataset provides the total area occupied and an estimate of the area covered by vegetation during the winter prior to sowing the spring crop. The detailed description of the variables is listed below:

• YEAR: Spring crop harvest year (2018 or 2019).
• INSEE: INSEE code of the municipality.
• CODE_CULTU: Code identification of the following spring crop as given in the French Registre Parcellaire Graphique.
• FOLLOWING_CROP: Translation of the CODE_CULTU code in English.
• TOTAL_AREA: Total area (in hectares) cultivated with this spring crop.
• COVERED_AREA: “Best” estimation of the area with winter cover crops before sowing this spring crop (in hectares).
• COVERED_AREA_MIN: Minimum estimation of the area with winter cover crops before sowing this spring crop (in hectares).
• COVERED_AREA_MAX: Maximum estimation of the area with winter cover crops before sowing this spring crop (in hectares).
• DEP: Department number.
• REG: Region number.

## 2 Experimental Design, Materials and Methods

This dataset provides an estimate of winter soil cover before spring crops at municipality level for mainland France for two years (2018 and 2019). It has been used to evaluate the effect of crop rotation [1] or soil and climatic conditions [2] on the adoption of winter cover crops in France.

The procedure for assessing winter soil cover was as follows:

1. Definition of the borders of plots with spring crops

For France, the limits of the plots declared within the context of the European Common Agricultural Policy are stored in a vector file known as the “Registre Parcellaire Graphique” [3,4]. For each year, this file indicates the type of crop grown on each plot. it was thus possible to select only the plots with spring crops with the variable “CODE_CULTU” of the attributes table, which gives the identification of each crop with a three letter code. To facilitate future use of this dataset, a "FOLLOWING_CROP" column has been added here to give the detailed name of each crop in English. Compared to the original “Registre Parcellaire Graphique” files, a 20 m negative buffer was applied to the borders of each plot to avoid edge effects due to sensor resolution or ground geolocation uncertainty during the soil cover detection stage.

2. Estimation of winter soil cover using Sentinel-2 multi-spectral images

Soil cover rate during the winter prior to sowing spring crops was estimated from the computation of the Normalized Difference Vegetation Index (NDVI) from multi-spectral images. The NDVI is calculated as follows:

(1) $$NDVI=\frac{\rho_{NIR}-\rho_{Red}}{\rho_{NIR}+\rho_{Red}}$$

Where ρ_NIR_ and ρ_Red_ stand for near infrared and red reflectance, respectively.

**Fig. 1:**
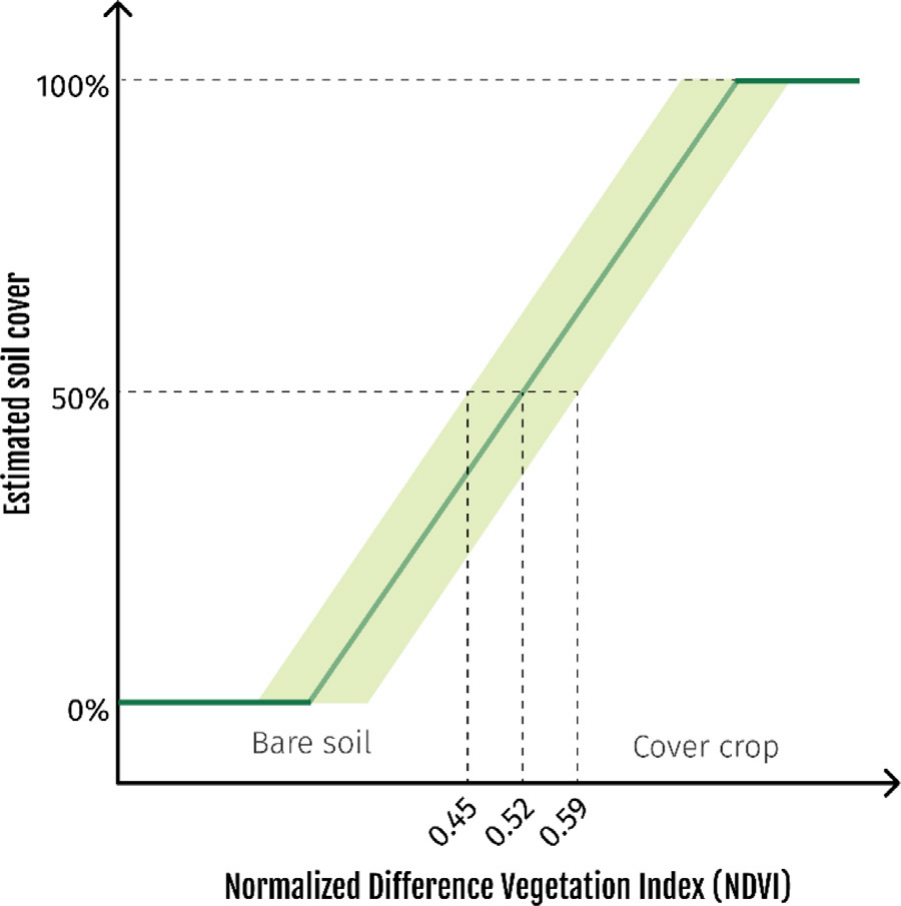
Estimation of soil cover by vegetation from the Normalized Difference Vegetation Index (NDVI). Based on the literature, a 50% soil cover corresponds to a threshold NDVI value between 0.45 and 0.59.

Because of the way it is calculated (Eq. (1)), the NDVI values are between −1 and 1. A bare soil has an NDVI value of about 0.2, and this value will increase with the development of the vegetation cover (up to NDVI values that can reach 0.9 for crops with high biomass). The NDVI is especially appropriate for monitoring winter soil cover because soil cover has a linear relationship with NDVI (Fig. 1), whereas it is more difficult to distinguish between high and very high crop biomass because the NDVI tends to saturate once the soil is completely covered by vegetation.

Yet soil characteristics, such as color or moisture, and crop residues influence NDVI measurements, especially in the early stages of crop development, when the soil is still poorly covered. In order to limit these effects, it was therefore considered that soil cover had to exceed 50% for a plot to be considered as covered by vegetation.

The linear relationship between NDVI and soil cover is well established in the literature and the NDVI values for 50% soil cover are similar across species [5,6], such as wheat [7,8] or soybeans [9] for example. The values reported by the studies are also similar for any source of multispectral images, from field sensor [10] to satellite images [11,12]. Overall, the NDVI values corresponding to a soil cover of 50% are between 0.45 and 0.59 (Fig. 1). Thus, to allow for sensitivity and uncertainty analyses for future studies that may be conducted using these data, three estimates are given: best estimate (column ”COVERED_AREA”, corresponding to the NDVI threshold of 0.52), minimum (“COVERED_AREA_MIN”, for a NDVI threshold of 0.59) and maximum (“COVERED_AREA_MAX”, for a NDVI threshold of 0.45).

The NDVI computation was carried out through the Google Earth Engine platform [13], using Sentinel-2 multispectral images at 10 m spatial resolution [14], corrected to surface reflectance using Sen2Cor [15]. Two levels of filters were applied to remove invalid observations [16]. First, for the study period, only the least cloudy images (20% threshold) were selected. Then, a second filter at the pixel scale was applied to remove observations identified as clouds, shadows or snow (using the Scene Classification map provided with Sentinel 2 observations). An example summarizing the code used can be found in the GitHub repository associated with this dataset [16].

**Fig. 2:**
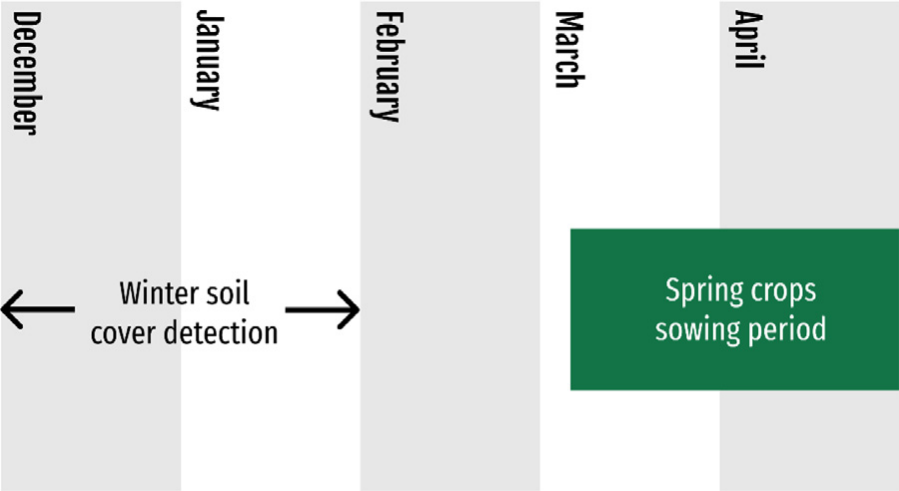
Timeline for cover crop detection.

For each year, winter soil cover monitoring was carried out for two months (December and January) during the winter before sowing the spring-sown crop (Fig. 2). December was chosen as the beginning of the study period to limit the risk of detecting unharvested spring-sown crops on the plots, such as grain maize or sugar beet that can be harvested late in the year. January was chosen as the end of the study period because some spring-sown crops, such as peas, can be sown as early as February. Furthermore, if a cover crop was present on the plot, it must have been already detected in December or January. For most plots, several NDVI values could be calculated for the study period. In this case, the maximum NDVI value was compared to the thresholds used to assess a soil cover rate above 50%.

Finally, results were aggregated at the municipality level. In the dataset, the “INSEE” column gives the INSEE code that identifies each municipality. For each crop and for each municipality, the total area occupied by the crop (in hectares) is specified, as well as three estimates (also in hectares) of the area covered, corresponding to the three NDVI thresholds defined above. For each crop and municipality combination, the given year corresponds to the year of harvest of the following spring crops (e.g. the year 2019 refers to the soil cover in December 2018 and January 2019).

## Ethics Statements

This work does not involve any type of human studies, animal studies, or data gathered using social media.

This manuscript adheres to ethics in publishing standards.

## CRediT authorship contribution statement

**Benjamin Nowak:** Conceptualization, Methodology, Data curation, Writing – original draft, Writing – review & editing. **Audrey Michaud:** Conceptualization, Writing – review & editing. **Gaëlle Marliac:** Conceptualization, Writing – review & editing.

## Declaration of Competing Interest

The authors declare that they have no known competing financial interests or personal relationships that could have appeared to influence the work reported in this paper.

## Data Availability

Dataset about the adoption of winter cover crops at the municipality level for mainland France (Original data) (GitHub).
